# Eye-tracking as a measure of receptive vocabulary in non-verbal children with cerebral palsy

**DOI:** 10.1371/journal.pone.0325183

**Published:** 2025-06-03

**Authors:** Susheel Joginder Singh, Suvalaxmi Raman, XinTong Lim, Vinitaa Rajakumar, Rogayah A. Razak, Shin Ying Chu

**Affiliations:** 1 Faculty of Health Sciences, Universiti Kebangsaan Malaysia, Kuala Lumpur, Malaysia; 2 Faculty of Social Sciences and Liberal Arts, UCSI University, Kuala Lumpur, Malaysia; University of Education, Lahore, PAKISTAN

## Abstract

Children with cerebral palsy (CP) often struggle to participate in traditional language assessments due to their limited mobility, making it challenging for speech-language therapists (SLTs) to accurately assess their language abilities. In recent years, there has been evidence that eye-tracking is an effective way of measuring the receptive language abilities of children who demonstrate difficulties with traditional language assessments. This study aimed to (i) develop eye-tracking assessment materials based on a receptive vocabulary subtest of a Malay language assessment and (ii) evaluate the performance of children with CP on the receptive vocabulary assessment conducted via eye-tracking, compared to their performance on a traditional receptive vocabulary assessment. The first phase of the study focused on developing eye-tracking receptive vocabulary assessment materials from the Malay Preschool Language Assessment Tool and trialling the materials and assessment protocol. This phase involved 15 typically developing children aged 4–6 years. The finalized materials and protocol were administered to 15 children with CP in the second phase. Each child attended two assessment sessions: the first was a traditional receptive vocabulary assessment, and the second utilized eye-tracking technology. Children practiced eye-tracking through online games. Results showed that eight children with CP performed better in the eye-tracking assessment, two scored similarly across both methods, and five scored lower during eye-tracking. The Wilcoxon Signed Rank Test conducted revealed no significant difference in scores across both assessment methods *(p* > .05). Furthermore, most children exhibited poor consistency in their scores across the two methods. These findings suggest that while some children with CP may benefit from receptive vocabulary assessments conducted via eye-tracking, no single assessment method is optimal for all children with CP. Instead, children with CP may benefit from a combination of assessment methods, including eye-tracking, to increase the accuracy of assessment results.

## Introduction

Cerebral palsy (CP) is a neuromotor disorder that affects the development of movement, muscle tone, and posture [1]. The pathophysiology is an injury to the developing brain that occurs during the pre-, peri, or postnatal period [1]. Many children with CP might have little control over their body movement and are dependent on assistance in most activities like eating, mobility, and play [2].

Children with CP experience speech, language, and communication difficulties of varying severity [1]. Some demonstrate dysarthria (speech disorder due to weakness of muscles of speech), leading to poor speech intelligibility or no speech [2]. In the presence of cognitive disturbances, children with CP may demonstrate difficulties with receptive and expressive language development [2] as language development appears to be highly correlated with non-verbal cognition. They also struggle with language development due to their lack of interaction with the world and reduced world experience [3]. Some children with CP might have good language comprehension abilities but cannot express themselves verbally due to dysarthria, leading communication partners to assume they have limited language comprehension [2–4].

Determining the language comprehension abilities of children with disabilities, including those with CP, especially those who are non-verbal, can be challenging [5,6]. It is important to know how much non-verbal children with CP can understand, as underestimation can lead to under-stimulation and, in turn, social isolation that can have a negative impact on a child’s well-being [5,7,8]. Traditional assessments of language comprehension/ receptive language, typically administered by speech-language therapists (SLTs), require the child to point at a picture that corresponds with the referent after it is named by the SLT (e.g., point to the ball). However, these traditional assessment methods might be unsuitable for children with CP with severe physical disabilities as well as children with limited behavior repertoires (e.g., autism) [9]. These children struggle to attend to the task, are unable to perform the pointing response, have limited understanding of the pragmatics of the test, and are socially unresponsive to the examiner [10]. These and other issues could affect the reliability and validity of the assessment and may lead to the underestimation of a child’s receptive language abilities [11]. SLTs may be unable to ascertain whether a child’s poor performance is due to the child’s limited language comprehension or inability to employ task-appropriate responding [11].

Previous researchers have employed various direct measures, such as standardized diagnostic assessments with adaptations [2], and indirect measures, like parental reports [12], to obtain more accurate results of the receptive language abilities of children with CP. Although adaptations are sometimes made to standardized assessments to facilitate responses from children with CP, these adaptations can inadvertently increase the cognitive load on the child [13]. Indirect measures, like parental reports, may have limitations, as parental reports can introduce biases, leading to both underestimates and overestimates of a child’s vocabulary knowledge.

As highlighted by Geytenbeek et al. [14], while various diagnostic tools exist to assess children’s receptive language abilities, very few are specifically designed for children with CP with severe motor impairments, highlighting the need for more accessible and accurate assessment tools. Over the years, researchers have sought alternative methods for assessing children’s language comprehension that do not require an understanding of test instructions or overt motor responses. Eye-tracking technology has emerged as a valuable tool for individuals with disabilities who may struggle to provide traditional responses due to compliance issues, motor challenges, cognitive limitations, or language barriers [10,11,15,16]. This technology employs a specialized camera to detect the position of a person’s eyes, tracking where they are looking on a computer screen.

Previous researchers [14,17,18] in their studies assessing children’s comprehension using standardized assessments that offer multiple response methods, including eye-gaze, scanning, and finger pointing, found that access methods do not significantly affect test results. This suggests that eye gaze is a valid method for assessing receptive language abilities of children with severe motor and speech impairments, enabling interventions to be grounded in reliable assessment data rather than assumptions or tests with compromised psychometric properties [13,17–19]. Although this technology has been successfully used to enhance communication for non-verbal children, its application in assessing receptive language for children with CP remains underexplored.

Malaysia is a fast-developing country in South-East Asia with a population of 32 million people. Although the field of speech-language pathology has been in Malaysia for more than four decades, Malaysian SLTs have yet to adopt eye-tracking for assessing receptive language abilities in children with disabilities, continuing to rely on traditional assessment methods that may not accurately reflect a child’s true capabilities [20]. Furthermore, until recently, Malaysian SLTs have primarily depended on foreign standardized receptive language assessments, with a standardized assessment in Malay only becoming available in the past few years.

This study aimed to develop eye-tracking assessment materials based on a receptive vocabulary subtest of a Malay language assessment and use these materials to assess non-verbal children with CP who face challenges with traditional assessment methods. The specific objectives of the study were: (a) to create materials for a Malay receptive vocabulary assessment to be administered via eye-tracking, and to test the assessment materials and protocol on typically developing children; (b) to determine the scores obtained by non-verbal children with CP during the eye-tracking assessment and compare them to scores obtained through traditional method; and (c) to determine the consistency of responses provided by children with CP across both assessment approaches.

## Materials and methods

This was a comparative study with purposive sampling. It was conducted in the Klang Valley of Malaysia. Ethics approval for this study was obtained from the author’s organization’s Human Research Ethics Body, JEP-2023–036. Recruitment and data collection for this study were conducted from 1 July 2023–30 September 2024.

### Participants and recruitment

This study involved 15 typically developing children and 15 children with CP. The inclusion criteria for typically developing children were as follows: (a) aged 4;0–6;11 years (based on the age requirement of the Malay Preschool Language Assessment Tool, MPLAT), (b) no language delays or difficulties (as reported by parents), (c) no reported hearing problems or vision problems that have not been corrected with spectacles, (d) no experience with eye-tracking in any form, and (e) have Malay as their first language. Typically developing children were recruited by contacting staff members at the authors’ organization and inviting those with children who met the inclusion criteria to participate in the study. Several kindergartens in the Klang Valley were also approached for participant recruitment, and advertisements about the study were posted on social media pages.

The inclusion criteria for children with CP were as follows: (a) aged 7–12 years old (based on the age recommendation of the MPLAT for children with communication disorders), (b) severe physical disabilities as determined by Levels 4 and 5 on the Gross Motor Functioning Classification System (GMFCS) and Manual Ability Classification System (MACS), (c) no reported hearing problems or vision problems that have not been corrected with spectacles, (d) no experience with eye-tracking in any form, (e) had not participated in any form of AAC intervention where eye gaze was the focus of intervention, (f) non-verbal communicators (have five or fewer words that were spoken consistently), and (g) have Malay as the family’s main spoken language at home. Children with CP were recruited from several early intervention centres in the Klang Valley.

Staff members at the kindergartens and early intervention centres were informed about the study and the inclusion criteria. They identified suitable participants and provided the information sheet and consent forms to the children’s parents, who then returned the signed consent form directly to the researcher. Parents provided consent for their children.

Twenty children with CP were recruited for the second phase of the study. Out of these 20, five children had to be excluded because of the lack of eye gaze on the screen, which prevented calibration of the eye-gaze equipment.

### Materials and equipment

#### Malay Preschool Language Assessment Tool (MPLAT).

MPLAT [21] is a standardized language assessment tool for Malay language speakers. It consists of six subtests, which are receptive picture vocabulary, grammatical understanding, sentence repetition, referential meaning, relational meaning, and early literacy skills. For this study, the receptive picture vocabulary subtest, which consists of 40 items, was used.

#### PCEye (Tobii Dynavox).

**Fig 1:**
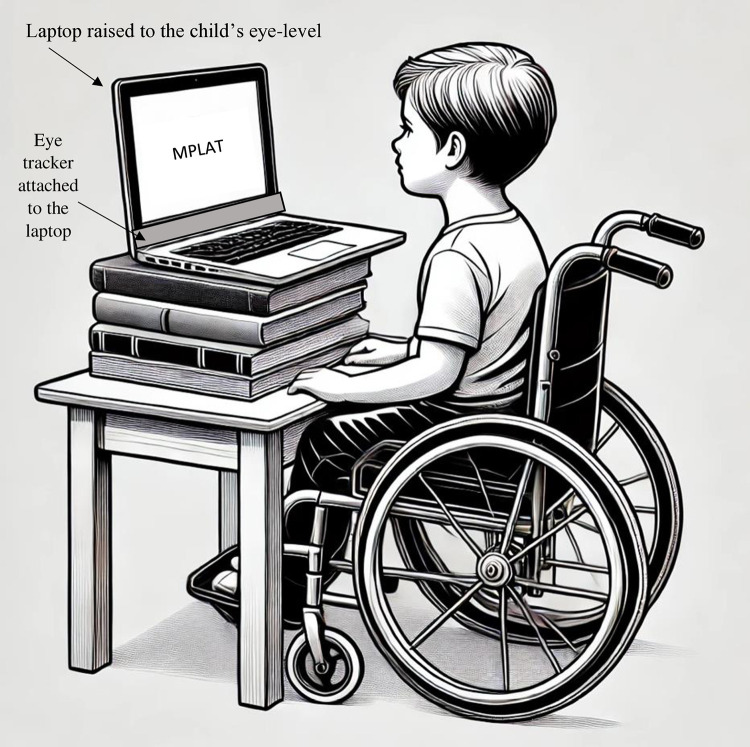
Setup of the laptop and eye-tracker during the eye-tracking assessment.

The PCEye, a portable eye-tracking device, was used for this study. It was connected to a laptop via a standard USB-2 cable. Participants’ eyes need to be 50–95 cm from the screen. To facilitate direct eye contact with the display, the screen’s position was carefully adjusted (see Fig 1). Through its software, TDControl, the PCEye projects an eye-gaze fixation arrow on the screen, indicating where participants are looking and enabling eye-fixation functions akin to mouse clicks. As a child scans the screen, the arrow moves, and when the child fixates on an area of interest (AOI) for a designated duration, it registers as a mouse click. The PCEye is particularly beneficial for children with physical disabilities, allowing for head movement without compromising accuracy or disrupting the eye-tracking session. Each participant underwent a calibration process tailored to their individual fixation durations (ranging from 0.8 to 2.5 seconds) to facilitate deliberate choices. Calibration involved tracking a small dot (1 cm radius) displayed on the screen for each child during every session.

#### Magic Eye FX.

**Fig 2:**
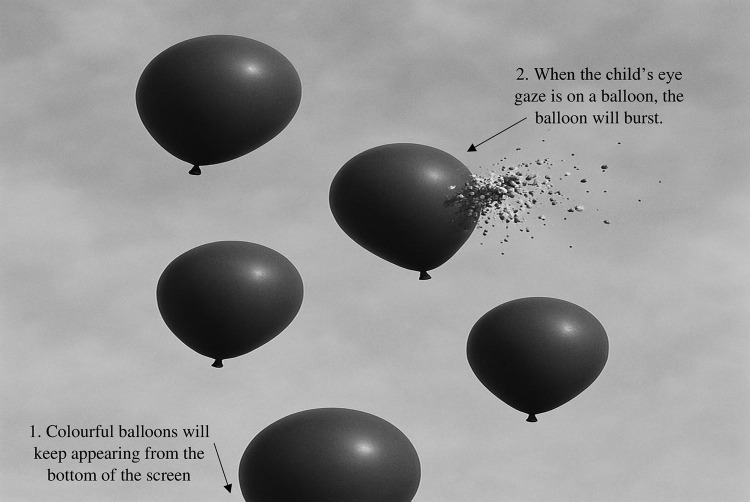
One of the games in Magic Eye FX.

The Magic Eye FX is a software that allows children to learn eye-gaze through games at five advancing levels of exploration: Level 1 Screen Exploration, Level 2 Object Interaction, Level 3 Region Precision, Level 4 Active Exploration, and Level 5 Controlled Targeting. The games enable gradual learning of eye fixation used to control the computer and make choices (Fig 2). At the simpler levels, the games gave visual and auditory feedback whenever the child focused his/her gaze on any part of the screen. At the more challenging levels, successful gameplay necessitated focusing on particular visual stimuli or selecting between different options by directing the gaze.

### Procedure

**Fig 3:**
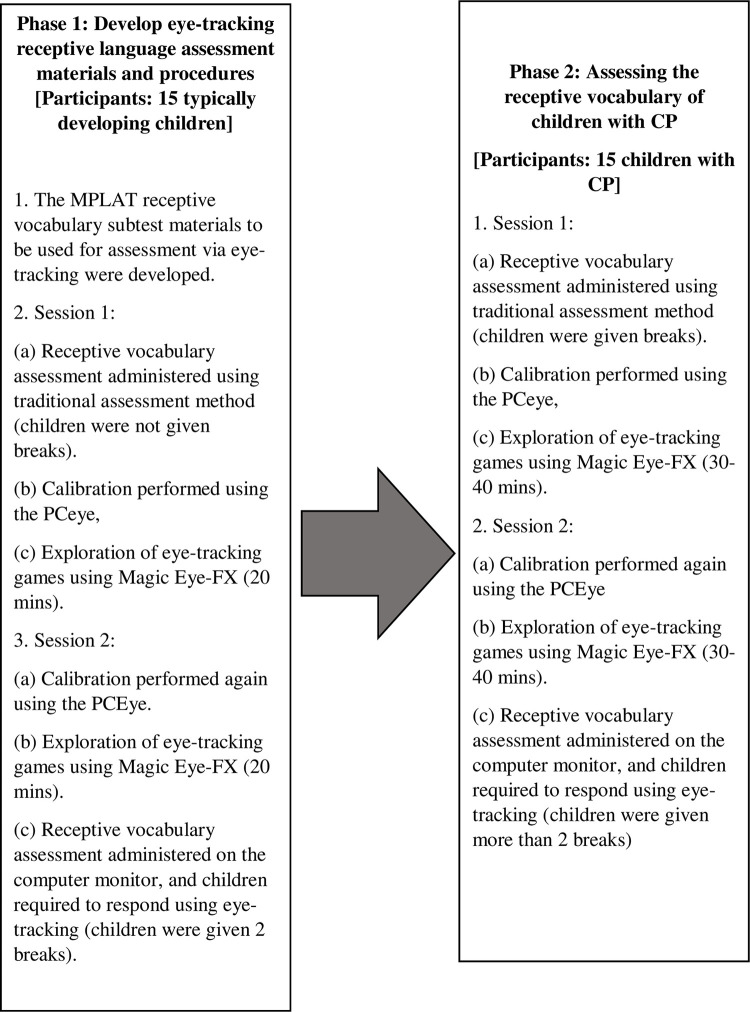
The study procedure.

This study had two phases (Fig 3) and was conducted in a therapy room with a double-sided mirror at the university clinic.

#### Phase 1: Develop the eye-tracking receptive language assessment materials and procedure.

Firstly, materials for the receptive language assessment to be conducted via eye tracking were developed. All pages from the MPLAT receptive picture vocabulary subtest were scanned and saved on a computer. Each page consists of four pictures presented in a 2x2 grid, and each picture was cropped and saved individually. Then, all the pictures were saved into a 2x2 grid in Google Forms, where the pictures could be selected by using the click of a mouse or eye-tracking (by fixating on a picture for a sufficient amount of time to indicate a mouse click and selecting that item). The size and arrangement of the pictures were the same as those in the MPLAT picture stimulus book.

The first phase of the study involved 15 typically developing children. The children attended two sessions. During the first session, the receptive picture vocabulary subtest from the MPLAT was administered using traditional assessment methods based on the guidelines outlined in the manual. This took approximately 15–20 minutes.

Next, eye-tracking technology was introduced. Children were seated in front of a laptop adjusted to their eye level, and calibration was conducted using the PCEye. This process required the children to track a moving dot on the screen, further confirming their visual capabilities. Calibration data for each child was saved in the system for future visits. A 9-point calibration was performed, and all the children achieved good results.

This was followed by the use of the Magic Eye-FX software, whereby children were able to explore the use of eye-tracking through games. The researchers selected several games from the Magic Eye-FX based on the different levels of difficulty and allowed the children to play. Each child was given about 20 minutes to play the games via eye-tracking. The first session took approximately an hour.

In the second session, calibration was again performed using the PCEye. Following calibration, the children spent about 20 minutes exploring eye-tracking technology through games using the Magic Eye-FX software. After a 10-minute break, the receptive picture vocabulary subtest from MPLAT was administered on the laptop. During this task, children were required to respond using eye-tracking, selecting the correct answer by fixating on an area of interest long enough to register a mouse click. To minimize fatigue, children were given two breaks during the assessment. Overall, the second session lasted approximately 70–90 minutes.

The researchers, along with two independent SLTs experienced in working with children with CP and using MPLAT, convened to discuss the findings from Phase 1 and the necessary adjustments to the assessment protocol for Phase 2. They decided to make several changes to the assessment protocol: (i) providing breaks for children with CP during the traditional assessment, (ii) allowing more than two breaks during the eye-tracking assessments based on individual needs to prevent over-exhaustion, and (iii) allocating 30–40 minutes for children to explore eye-tracking through games in each session as previous researchers have cautioned that children with CP took twice as long as typically developing children to complete the games [22].

#### Phase 2: Assessing the receptive vocabulary of children with CP.

This phase involved 15 children with CP. The assessment protocol was the same as Phase 1 with some changes to the assessment protocol as stated previously. The children with CP were seated in the most suitable adaptive seating available or their wheelchairs during both sessions. For four children with CP, calibration needed to be performed several times to achieve good results because these children kept looking away from the screen. There were five children who were recruited for the study who had to be excluded because they were unable to focus on the screen, preventing calibration of the PCEye for them.

### Data analysis

A Wilcoxon Signed-Rank Test was conducted to determine whether there was a statistically significant difference between scores across the two assessment methods. A nonparametric test was utilized as the data failed to meet the assumptions required for a parametric test, including a small sample size and non-normal distribution of differences in scores between the two assessment methods.

To identify how consistently a child provided the same answer to a question across the two assessments, the agreement was calculated using Cohen’s Kappa. Interpretation of the Kappa coefficient was based on the work of McHugh [23]: 0 = poor,.01 to.20 = slight,.21 to.40 = fair,.41 to.60 = moderate,.61 to.80 = substantial, and.81 to 1.00 = almost perfect.

## Results

A total of 15 typically developing children participated in Phase 1 of the study to trial out the MPLAT receptive language assessment via eye-tracking. Among these 15 children, 6 (35%) were 4 years old, 5 (30%) were 5 years old, and 4 (30%) were 6 years old.

The children were administered the MPLAT receptive language assessment via the traditional method and eye-tracking, and the results are presented in Table 1. The highest scores obtained by children during the assessment conducted via the traditional method and eye-tracking were 36 and 37, respectively.. The lowest scores obtained by children during the assessment conducted via the traditional method and eye-tracking were 21 and 24, respectively. The mean scores obtained during the assessment conducted via traditional method and eye tracking were 26.5 (*SD* = 4.44) and 29.07 (*SD* = 4.28), respectively.

**Table 1:** Scores obtained by typically developing children during assessment and consistency of response across assessments.

Child	Gender	Age	Total Score (out of 40)		Cohen’s Kappa (κ)
			Traditional	Eye tracking	
TD 1 (16)	M	4;00	25	26	0.646
TD 2 (19)	M	4;01	22	24	0.798
TD 3 (4)	F	4;01	28	27	0.649
TD 4 (13)	M	4;03	21	24	756
TD 5 (14)	F	4;06	22	24	0.931
TD 6 (11)	F	4;11	23	28	0.730
TD 7 (15)	F	5;00	27	28	0.828
TD 8 (20)	M	5;00	24	29	0.831
TD 9 (7)	M	5;00	23	25	0. 731
TD 10 (8)	F	5;02	36	35	0.897
TD 11 (2)	F	5;05	33	34	0.930
TD 12 (9)	F	6;02	26	32	0.751
TD 13 (6)	M	6;03	29	33	0.709
TD 14 (3)	M	6;05	27	30	0.856
TD 15 (12)	M	6;06	32	37	0.721

The scores obtained by the children in this study from the traditional and eye-tracking methods were compared to the mean score and SD stated in the MPLAT manual (see Table 2). For the traditional method, 11 (85%) children obtained scores similar to or above the mean scores of their age group as stated in the MPLAT. Four children obtained scores below the mean but within one SD of the mean score, indicating no concern. Whereas for the eye-tracking method, all (100%) children scored above the mean score.

**Table 2:** Mean scores and standard deviation for the receptive vocabulary sub-test of MPLAT.

Age group	Mean	SD	Standard error	Min	Max
4;0-4-5	19.4	4.0	0.5	4	25
4;6−4;11	22.1	2.6	0.3	15	31
5;0-5;5	24.0	4.2	0.5	7	35
5;6−5;11	26.0	3.6	0.4	18	35
6;0-6;5	28.8	3.3	0.4	21	37
6;6−6;11	31.6	3.1	0.4	24	38

Thirteen children (85%) scored higher during the assessment conducted via eye-tracking compared to the traditional method, and two children (T3 and T10) scored lower during the assessment conducted via eye-tracking compared to the traditional method. A Wilcoxon signed-rank test was conducted to compare scores between the traditional (Mdn = 26) and eye-tracking (Mdn = 29) assessment methods. The test revealed a significant difference in scores, W = 6.00, Z = −3.085, p = .002, whereby higher scores were obtained during eye-tracking, with a large effect size (r = −0.86). The 95% confidence interval for the median difference between the traditional method and eye-tracking was [−1.0,3.0].

Cohen’s Kappa was calculated to identify how consistently a child provided the same answer to a question across the two assessments. The results (see Table 1) revealed that nine (60%) children showed substantial agreement, and six (40%) children showed almost perfect agreement. The mean kappa value was 0.736, and thus it can be concluded that the children showed more than substantial agreement between traditional and eye-tracking methods.

Fifteen children with CP were involved in the second phase of the study. The background information of these children is presented in Table 3. These children ranged from 6 to 12 years old. The children were all at Levels 4 and 5 of the MACS and GMFCS. Eight children did not have vision impairment, while the others had their vision corrected using eyeglasses and wore eyeglasses during this study. All children were non-verbal communicators (had five or fewer words that were spoken consistently).

**Table 3:** Background information of children with cerebral palsy, scores obtained during assessment, and consistency of response across assessments.

Child	Age	Gender	GMFCS^a^ level	MACS^b^ level	Total Score (out of 40)		Cohen’s Kappa (κ)
					Traditional	Eye Tracking	
1	6;06	Male	5	5	20	20	0.828
2	6;06	Female	5	5	32	21	0.563
3	7;06	Male	5	5	21	10	0.286
4	6;09	Male	5	5	0	36	0
5	12;04	Female	5	5	15	24	0.661
6	11;06	Male	4	4	2	10	0.033
7	10;06	Male	4	4	11	14	0.209
8	9;04	Female	5	5	0	34	0
9	11;03	Female	4	4	22	12	0.462
10	12;02	Female	4	5	23	5	0.368
11	9;04	Male	5	5	22	11	0.267
12	8;09	Male	5	5	16	24	0.656
13	8;04	Female	5	5	15	37	0.243
14	7;04	Male	4	5	8	15	0. 0.265
15	10;02	Female	4	5	23	23	0.864

^a^Gross Motor Functioning Classification System

^b^Manual Ability Classification System

The scores obtained by children with CP using the traditional assessment method and eye-tracking method are presented in Table 3. The highest scores obtained during the assessment via traditional and eye tracking methods were 32 and 37 respectively. The lowest score obtained was 0 during the assessment via the traditional method and 5 during eye-tracking assessment. The mean scores obtained by children with CP for the assessment conducted via traditional method and eye tracking were 15.33 (*SD* = 9.84) and 19.73 (*SD* = 10.0) respectively.

Eight children (53.3%) obtained higher scores during the assessment conducted via eye-tracking compared to the traditional method. Two children (C4 and C8) obtained 0 during the traditional assessment and 36 and 34 respectively during the assessment via eye-tracking. Five children obtained higher scores during the assessment conducted via the traditional method compared to the eye tracking method. Two children obtained the same scores for both assessment methods. A Wilcoxon signed-rank test was conducted to compare scores between assessment using traditional method (Mdn = 15) and eye-tracking (Mdn = 14). The test did not reveal a statistically significant difference between the two assessment methods, W = 40.00, Z = 0.38, p = .70, with a small effect size (r = 0.10). The 95% confidence interval for the median difference was [−6.75, 12.75].

Cohen’s Kappa was calculated to identify how consistently a child provided the same answer to a question across the two assessments. The results (see Table 3) revealed that two children showed almost perfect agreement, two showed substantial agreement, two showed moderate agreement, six showed fair agreement, one showed slight agreement, and two showed poor agreement. The mean kappa value was 0.252, indicating that the children showed slight agreement between traditional and eye-tracking methods. Two children (C1 and C15) provided identical responses in both methods for all questions and their consistency of response across both methods was almost perfect. The two children (C4 and C8) who obtained 0 during the assessment via the traditional method had a consistency score of 0.

Overall, findings from this study showed that children with CP generally scored higher using the eye-tracking method compared to the traditional method, though the difference was not statistically significant. Consistency of responses between the two methods varied, with most children showing slight to fair agreement, and only two demonstrating almost perfect agreement.

## Discussion

The first phase of this study aimed to develop materials for a Malay receptive vocabulary assessment to be administered via eye-tracking and trial the assessment materials and protocol on typically developing children. All the typically developing children were administered the MPLAT receptive vocabulary assessment twice, once via the traditional method and once more via eye-tracking. The scores obtained by the children via both methods were within the range of scores of the MPLAT, suggesting that the administration of the MPLAT via eye-tracking was a valid form of assessment. There was also substantial consistency in scores between the two assessment methods, implying that the children performed similarly. These findings indicate that access methods did not influence the children’s performance, a finding similar to Fiske et al. [17]. Furthermore, the substantial response consistency between both assessment methods suggests that the children knew the answers for the items and were not fixating on pictures only because of interest, as cautioned by Borgestig et al. [8]. According to Brady et al. [11], eye gaze responses that do not match the pointing response reflect problems in the procedure, which was not observed in this study, given the consistency of responses across both methods.

However, there was a significant difference in children’s scores across the two assessment methods, with more children obtaining higher scores during the assessment conducted via eye-tracking than the traditional method. According to Chita-Tegmark et al. [24], online eye gaze measures of receptive language have minimal response demands on a child, as it only requires them to look at the image names rather than a complex non-verbal response of pointing which sometimes leads to young children not being able to exhibit the extent of their knowledge. Chita-Tegmark et al. [24] suggested that online eye-gaze measures of receptive language are more sensitive to children’s word knowledge due to the low task demand on children. The difference in scores obtained by children in this study across the two assessment measures could have been because the children were more focused during the assessment via eye-tracking, paying more attention to the ‘looking’ rather than the ‘pointing’ action [25]. Alternatively, as suggested by Fernald et al. [26], children require different reaction times based on their age and familiarity with the words, and the eye-tracking device visualized the children’s eye gaze pattern, allowing examiners to know that the child required more processing time. It could be that during the assessment via eye-tracking, the children who spent longer looking at the four answer options without fixating their eye-gaze were unintentionally given more processing time compared to the time given to them during the traditional assessment, as visualization of processing did not show during the traditional assessment.

All the children in this study had no experience using eye-tracking devices; thus, letting them familiarize themselves with eye-tracking using game-like activities was important before conducting the assessment via eye-tracking [9]. According to Borgestig et al. [8] and Bekteshi et al. [22], children’s eye-gaze performance improves over time and with experience. In this study, it was observed that the children developed eye-tracking skills when given a period to familiarize themselves with the eye-tracking device. Through the games played via Magic-Eye FX, the children were able to understand the concept of gaze response, whereby something happens when they look at the screen during the games played. The children were also able to grasp the concept of fixation, whereby they needed to look long enough for something to happen on the screen while playing the games. This concept was the same as looking at the pictures with enough fixation time to indicate the selection of the pictures during the assessment.

Following the analysis from Phase 1 of the study, it was ascertained that the online MPLAT material that was developed and the assessment protocol were suitable to be used for children with special needs. In the second phase of the study, 15 children with CP were administered the MPLAT receptive vocabulary assessment using the traditional method and eye tracking.

The results indicated that, as a group, children with CP showed no significant differences in scores between the two assessment methods, suggesting that they did not perform better on one over the other. The considerable heterogeneity among the participants in terms of motor abilities, intellectual functioning, and attention span likely contributed to varied responses during the assessments. Notably, three children with CP achieved scores above 85% using eye-tracking, yet their consistency was low due to poor performance in traditional assessments, with two of them scoring zero. These three children were classified as level V on the GMFCS and MACS scales, which hindered their ability to participate in traditional assessments due to limited hand movement. Eye-tracking technology allows children with severe motor impairments to demonstrate their abilities, which may be overlooked in conventional assessments due to physical constraints [19]. Therefore, it is essential to provide reliable response modalities for assessing children with significantly limited motor function, ensuring that their capabilities are recognized rather than overshadowed by their physical limitations [27]. The results from these three children suggest that eye-tracking assessments may be more effective for some individuals with severe physical disabilities who can focus on the task at hand.

Out of the eight children with CP who scored higher on the eye-tracking assessment, three obtained scores of 15 or less on both the eye-tracking and traditional assessments. These children demonstrated low consistency in their scores across both methods, suggesting they may have limited receptive vocabulary. The poor performance in both assessments indicates to SLTs that these children struggle with vocabulary, rather than simply having difficulty pointing during the evaluation. It’s possible that some of these children also experience a degree of intellectual disability, as studies have shown a positive correlation between intellectual functioning and language comprehension in children with CP [18]. Additionally, children with CP may face delayed language development and restricted vocabulary due to limited interaction with their environment, stemming from their physical challenges [28].

Two children achieved identical scores across both assessment methods and exhibited high consistency in their results. For these children, the different access methods did not appear to impact their performance, mirroring findings from Fiske et al. [17]. This once again underscores the heterogeneity among children with CP, as some do not struggle with any assessment methods. However, it is worth noting that for these children, the traditional assessment took significantly longer than the eye-tracking assessment, and they required frequent breaks. This observation emphasizes the importance for SLTs to remain attentive to each child’s needs during assessments to prevent excessive fatigue.

Five children scored lower on the eye-tracking assessment compared to the traditional method, with all five achieving scores of 21 and higher in the traditional assessment and 21 or lower in the eye-tracking assessment. During the eye-tracking evaluation, these children struggled with attention and required more breaks as the assessment progressed. Despite achieving good calibration and participating well in the eye-tracking games, their performance declined over time. Research indicates that children who are fidgety and have poor attention can yield low-quality eye-tracking data [10,29]. Thus, the lower scores during the eye-tracking assessment may reflect a lack of focus and interest in the task. It could also be that the task was too long for these children and the assessment needed to be broken up across several days, as done by Ahoniska Assa [9] in their study involving children with Rett’s syndrome, to account for exhaustion.

While specific studies focusing on the use of eye-tracking technology to measure vocabulary in children with CP are limited, several research efforts have successfully explored its application in assessing language comprehension in other populations of children with developmental disabilities [10,11]. These studies also revealed significant variability in receptive language abilities among children, highlighting how heterogeneity in behavioural challenges, task engagement, motivation, attention profiles, and cognitive impairments affected their performance [10]. Similar to the current study, previous researchers emphasized that no single assessment or response method is suitable for all children, underscoring the need for individualized approaches to identify the language comprehension of minimally verbal children [10].

### Clinical implications

Children with CP remain one of the most challenging populations for SLTs to assess, as they often struggle to respond to traditional language assessments. This can lead to an underestimation of their receptive language abilities. The findings from this study suggest that eye-tracking may serve as an effective alternative assessment method for some children with severe physical limitations. This approach enables SLTs to gain deeper insights into their receptive language skills, which can ultimately inform the development of more tailored therapy goals. For some children with CP, eye-tracking serves as a supplementary assessment alongside traditional methods to verify consistency in findings and to ensure that poor performance is due to limited vocabulary and not physical limitations. Therefore, integrating eye-tracking into clinical practice may be essential for SLTs as it provides a more inclusive and unbiassed approach to assessing receptive vocabulary for this population.

Results obtained for children with CP in this study varied significantly from one individual to another. These findings suggest that there is no one assessment method that is suitable for all children. Similarly, a study done by Ward et al. [30] on children with Rett Syndrome revealed that generalization on the best method of assessment for all children should not be done as children respond differently to various assessment methods. Instead, each child’s needs, preferences, and response style should be taken into consideration by SLTs when deciding upon an assessment [30].

Eye tracking is not only a viable method of assessment for children with CP, but it also provides them with opportunities to learn and communicate (i.e., to access their augmentative and alternative communication system). Several studies on eye-tracking involving children with CP have highlighted that structured and tailored eye-tracking intervention can improve the performance of children with CP on eye-tracking [22,31]. Therefore, introducing eye-tracking technology early to children with CP early might maximize its benefits.

### Limitations and future research

This study has several limitations that may have affected the results. First, the small sample size of both typically developing children and children with CP limits the conclusions that can be made from this study. Future research should aim to include a larger sample to increase the statistical power of the study, making it more likely to detect meaningful differences and trends. With a larger and more diverse group of participants, researchers could better explore how individual factors, such as the type of CP, age, and cognitive abilities, might influence the effectiveness of eye-tracking. Expanding the participant pool would also allow for more reliable comparisons between assessment methods, ultimately helping to determine whether eye-tracking offers distinct advantages in capturing the receptive language skills of children with CP.

Another limitation is the limited practice time given to the children before the eye-tracking assessment. For some participants, additional practice could have improved their performance. Research indicates that children with CP who have prior experience with eye-tracking tend to perform better than those without such experience [8,22]. Future research should explore ways to optimize eye-tracking assessments, such as adjusting task length or incorporating breaks, to enhance engagement and accuracy.

## Conclusion

Children with CP frequently experience language difficulties of varying severity. Given the challenges these children face with traditional language assessments, SLTs must provide alternative access and response methods during receptive language evaluations to obtain more accurate insights into their abilities. Overall, findings from this study highlight the complex interplay between motor abilities, intellectual functioning, and attention span in shaping the performance of children with CP during vocabulary assessments. While eye-tracking provided a viable alternative for some children with severe motor impairments, enabling them to demonstrate receptive vocabulary skills that were otherwise undetectable through traditional methods, it was not universally advantageous for all participants. Some children exhibited lower scores due to attentional difficulties and fatigue. These findings underscore the need for SLTs to choose assessment methods based on the child’s need, as no one assessment method might be suitable for all children with CP. Additionally, comparing results across different access methods helps SLTs determine whether a child’s poor performance is due to difficulties with the response method or limitations in language comprehension. Accurate assessment results will enable SLTs to plan intervention goals that promote language development in children with CP.

## Supporting information

S1 Dataset Dataset of assessment scores for typically developing children and children with cerebral palsy. (XLSX)
